# NIA DIVISION OF AGING BIOLOGY

**DOI:** 10.1093/geroni/igad104.0833

**Published:** 2023-12-21

**Authors:** Ronald Kohanski

**Affiliations:** National Institute on Aging, Bethesda, Maryland, United States

## Abstract

Dr. Kohanski will discuss research priorities for the NIA Division of Aging Biology. Dr. Kohanski will also be available for small group discussion.

